# Prions in Muscles of Cervids with Chronic Wasting Disease, Norway

**DOI:** 10.3201/eid3102.240903

**Published:** 2025-02

**Authors:** Tram T. Vuong, Federico A. Cazzaniga, Linh Tran, Jørn Våge, Michele Di Bari, Laura Pirisinu, Claudia D’Agostino, Romolo Nonno, Fabio Moda, Sylvie L. Benestad

**Affiliations:** Norwegian Veterinary Institute, Ås, Norway (T.T. Vuong, L. Tran, J. Våge, S.L. Benestad); Fondazione IRCCS Istituto Neurologico Carlo Besta, Milano, Italy (F.A. Cazzaniga, F. Moda); Istituto Superiore di Sanità, Rome, Italy (M.D. Bari, L. Pirisinu, C. D’Agostino, R. Nonno); Università degli Studi di Milano (F. Moda)

**Keywords:** Prions, muscles, cervids, chronic wasting disease, lymph nodes, tissue distribution, protein misfolding cyclic amplification, Norway

## Abstract

Chronic wasting disease (CWD) is an emerging prion disease in Nordic countries and has been detected in reindeer, moose, and red deer since 2016. CWD sporadically detected in moose and red deer in 3 Nordic countries demonstrated pathologic and strain characteristics different from CWD in reindeer, including an unexpected lack of prions outside the central nervous system as measured by standard diagnostic tests. Using protein misfolding cyclic amplification, we detected prions in the lymphoreticular system of moose and red deer with CWD in Norway and, remarkably, in muscles of both of those species and in CWD-infected reindeer. One moose lymph node and 1 moose muscle sample showed infectivity when experimentally transmitted to bank voles. Our findings highlight the systemic nature of CWD strains in Europe and raise questions regarding the risk of human exposure through edible tissues.

Chronic wasting disease (CWD) is a fatal disease affecting several species of cervids (*1*,*2*). It belongs to the group of transmissible spongiform encephalopathies, including scrapie in sheep and goats, bovine spongiform encephalopathy (BSE) in cattle, and Creutzfeldt-Jakob disease in humans (*3*). Transmissible spongiform encephalopathies are caused by misfolded forms of the prion protein (PrP^C^), known as PrP^Sc^ (prions), which are thought to replicate in an autocatalytic process that converts PrP^C^ into PrP^Sc^ (*4*). The diseases are characterized by spongiform changes and the accumulation of PrP^Sc^ in the central nervous system (CNS).

Researchers first described CWD in North America in 1967, and evidence of the disease has since then inexorably expanded across 34 states in the United States and 4 provinces in Canada (*5*). CWD emerged for the first time in Europe in a wild reindeer (*Rangifer tarandus*) in Norway and, shortly thereafter, in 2 moose (*Alces alces*). As part of an extensive surveillance program in Norway, investigators have identified 21 reindeer, 13 moose, and 3 red deer (*Cervus elaphus*) as being infected with CWD. A 3-year surveillance program in countries in Europe that have wild reindeer or moose revealed CWD in 3 moose in Finland and in 4 moose in Sweden.

The origin of the CWD disease identified in Europe is not known. Increasing data showed that the prion strains found in cases from northern Europe were multiple and different from those in North America (*6*–*8*). The strain found in reindeer closely resembled the North America strains in terms of distribution of PrP^Sc^, first in the lymphoreticular system and later in the brain, and the contagious characteristics in the natural host. Nevertheless, the strain of CWD found in reindeer in Europe was not identical to the CWD characterized from North America (*9*,*10*). Furthermore, CWD strains from moose in Nordic countries demonstrated substantial differences when compared with North America strains. Those moose, primarily old animals with a sporadic geographic distribution in Norway, Finland, and Sweden, exhibited unique characteristics not previously documented, and their infections were proposed as sporadic CWD (*11*). Transmission studies in bank voles and in transgenic mice expressing cervid PrP revealed that CWD prions in moose clearly differ from CWD prions from both reindeer in Norway and from the North America isolates studied. Furthermore, researchers have observed PrP^Sc^ and strain variation among individual moose isolates (*9*,*10*,*12*,*13*). Researchers studying CWD-affected moose and using traditional immunodetection tests (ELISA, Western blot [WB], and immunohistochemistry) detected PrP^Sc^ in the CNS and not in the lymphoreticular system (*13*,*14*). As in moose, PrP^Sc^ is not lymphotropic in red deer, but analysis on the first CWD-affected red deer in Norway revealed numerous characteristics that point to a unique CWD strain (*6*,*15*–*18*).

CWD has an incubation period of several years, during which time infected animals can shed prions in excreta, even before showing clinical signs (*19*–*22*). On the basis of investigations of sheep scrapie and cattle BSE, researchers have commonly hypothesized that CWD cases demonstrating a disease phenotype with general lymphoreticular involvement have higher potential to transmit the disease in field conditions (*23*). The anatomic distribution in the CNS and the lymphotropic properties of PrP^Sc^ distinguish the different prion strains (*24*,*25*). Those insights have inspired conjecture regarding a possible (albeit undetectable with conventional assays) accumulation of infectious prions in the peripheral tissues of the newly emerged strains in cervids in Norway, especially in the strains identified in moose and red deer. We used protein misfolding cyclic amplification (PMCA) and animal bioassay to assess the presence of CWD prions and their potential infectivity in lymph nodes, nerves, and muscles from CWD field cases in Norway. 

## Materials and Methods

### Animal Tissues

We detected CWD-affected animals (Table 1) through Norway’s surveillance program for CWD by using TeSeE (Bio-Rad Laboratories, https://www.bio-rad.com) or HerdChek (IDEXX, https://www.idexx.com) ELISA tests and confirmed results by TeSeE Western blot (Bio-Rad). We collected tissue samples from different parts of 2 reindeer, 4 moose, and 1 red deer by using disposable instruments to avoid cross contamination (Table 2). We used 3 reindeer, 3 moose and 1 red deer, all healthy and confirmed PrP^Sc^-negative, as negative controls.

**Table 1:** Information for animals included in a study of prions in muscles of cervids with CWD, Norway*

Species	ID number	Code	CWD genotype	Animal sex	Area detected	Diagnostic status†	
						Brain	Lymph node
*Rangifer tarandus*	17-CD20830	Reindeer A	A/A‡	M	Nordfjella	Positive	Positive
	18-CD3207	Reindeer B	A/B‡	F	Nordfjella	Negative	Positive
*Alces alces*	17-CD11399	Moose A	KK_109_	F	Lierne	Positive	Negative
	21-CD41	Moose B	KK_109_	M	Telemark	Positive	Negative
	19-CD24854	Moose C	QQ_109_	F	Sigdal	Positive	Negative
	22-CD29	Moose D	QQ_109_	F	Tynset	Positive	Negative
*Cervus elaphus*	17-CD14051	Red deer	QQ_226_	F	Gjemnes	Positive	Negative

*Animals detected through Norway’s CWD surveillance program. CWD, chronic wasting disease; ID, identification. †PrP^Sc^ detected by traditional diagnostic methods: ELISA, Western blot, and immunohistochemistry. ‡Nomenclature according to Güere et al. (*26*).

**Table 2:** Summary of PMCA amplification of prions in selected peripheral tissues from CWD-affected reindeer, moose, and a red deer with different strains in a study of prions in muscles of cervids with CWD, Norway*

Tissues or organs	Reindeer			Moose				Red deer
	A	B		A	B	C	D	
Lymph nodes								
Retropharyngeal	NA	NA		NA	Amp	NA	NA	Amp
Parotid	Amp	Amp		Amp, inf	NA	Amp	NA	NA
Mandibular	NA	NA		NA	NA	NA	No Amp	NA
Prescapular	Amp	Amp		NA	NA	No amp, no inf	Inc	No amp, no inf
Axillary	NA	NA		Amp	NA	NA	NA	NA
Popliteal	Amp	Amp		No amp, no inf	NA	No amp	No amp	NA
Peripheral nerves								
Brachial plexus	Amp	NA		Amp	NA	Amp	NA	Amp
Ischiadic nerve	Amp	Amp		Amp	NA	Amp	Amp	Amp
Optic nerve	Amp	NA		Amp	NA	Amp	NA	Amp
Muscles								
Masseter	Amp	Amp		Amp	Amp	Amp	Amp	Amp
Triceps brachii	Inc	No amp		Amp	Amp	Amp	Inc	Amp
Psoas	Amp	Amp		Amp	Amp	Amp	No amp	No amp
Longissimus dorsi	Amp	NA		Amp, no inf	Amp	Amp, no Inf	No amp	Inc
Semitendinosus	No amp	NA		No amp	Amp	Amp	No amp	No amp
Ocular	Amp	NA		Amp, inf	NA	Amp	NA	Amp
Lingual	Amp	Amp		Amp	NA	Amp	NA	Amp
Cardiac	Amp	Amp		NA	Amp	No amp	No amp	NA

*Amp, positive amplification; CWD, chronic wasting disease; inc, inconclusive results due to inconsistencies in the 2 laboratories; inf, positive infectivity in Bv109I voles; NA, not available/not tested; no amp, no amplification; no inf, no infectivity in Bv109I voles; PMCA, protein misfolding cyclic amplification.

### Tissues Preparation

We homogenized all biologic tissues in phosphate-buffered saline at 10% wt/vol and used grinding tubes with silica beads and TeSeE PRECESS 48 homogenizer (Bio-Rad). For the muscle and lymph node tissues, we applied 2 ribolysing cycles of 60 seconds ribolysis and 30 seconds rest to improve the homogenization quality. Except for the brain samples that were tested at 10^−4^ to 10^−8^ dilutions to assess PMCA sensitivity, we tested all samples undiluted.

### PMCA

Scientists performed PMCA in parallel in laboratories in Ås (Lab-Å) and in Milan (Lab-M). We prepared PMCA substrate by homogenizing bank vole brains (PRNP 109M; obtained from the Italian National Institute of Health, Rome) at 10% wt/vol in conversion buffer consisting of phosphate-buffered saline supplemented with a cocktail of 150 mM NaCl, 1% Triton X-100, and protease inhibitors (Roche, https://www.roche.com). We removed debris by centrifugation at 800 × *g* for 1 minute before aliquoting and storing the supernatant at −80°C. Before PMCA, we supplemented the substrate with 0.1 mg/mL heparin and 0.05% digitonin to increase the amplification efficiency. 

To run PMCA analyses, we added 10 µL of each sample to 90 µL of PMCA reaction substrate in a PCR tube with 3 poly(tetrafluoroethylene) beads (Marteau & Lemarié, https://www.marteau-lemarie.fr). We then subjected the mixtures to cycles of sonication (30 seconds at 230–250 watts) and incubation (29 minutes 30 seconds at 36°C–40°C) using a Q700 sonicator (QSonica, https://www.sonicator.com), in 96 cycles for 1 PMCA round. We employed a slightly different experimental setting in Lab-M: 20 seconds sonication at 150–170 watts and 29 minutes 40 seconds incubation at 37°C. When available, we analyzed equivalent tissues from CWD-negative animals in parallel as negative controls for the amplification. We performed a maximum of 6 PMCA rounds and evaluated the resulting PMCA products by WB.

### WB Analysis

#### Brain Isolates

We prepared and analyzed brain homogenates according to the instructions of the TeSeE Western blot kit (Bio-Rad), with a slight modification: we performed the electrophoresis using NuPAGE 12% Bis-Tris protein gels (Thermo Scientific, https://www.thermofisher.com). To distinguish the PrP^res^ (protease-resistant prion) types according to the differential proteinase K cleavage at the N terminus, we used 2 different antibodies: Sha31 antibody (TeSeE Western blot kit), which recognizes the epitope (aa 145–152) in the core protein; and monoclonal antibody (mAb) 12B2 (1:1000; Wageningen Bioveterinary Research, https://www.wur.nl), which binds the epitope (aa 89–93) situated at N-terminal of the prion protein.

#### PMCA Products

We performed WB analysis of the PMCA products by using a standard protocol, with slight differences between Lab-Å and Lab-M. We treated the amplified products with proteinase K (Sigma-Aldrich Solutions, https://www.sigmaaldrich.com) at a final concentration of 100 µg/mL (or 50 µg/mL) for 1 hour at 37°C. We halted digestion by adding Laemmli buffer and boiling the samples at 100°C for 5 minutes (or 10 minutes) before conducting sodium dodecyl sulfate–polyacrylamide gel electrophoresis by using NuPAGE 12% Bis-Tris protein gels (Thermo Scientific). We transferred proteins onto a polyvinylidene difluoride membrane and subjected them to immunodetection using the mAb Sha31clone, at a dilution of 1:10 (TeSeE Western blot kit [Bio-Rad]), or the mAb 6D11 clone (Biolegend, https://www.biolegend.com), at a dilution of 1:5000. We developed results by using SuperSignal West Pico plus chemiluminescent substrate (Thermo Scientific) or ECL Prime (GE Healthcare, https://www.gehealthcare.com) and visualized using Azure c280 (Azure Biosystems, https://azurebiosystems.com). We considered a sample positive upon detection of a pattern of 3 protease-resistant bands at expected electrophoretic mobility within 6 rounds of PMCA. We considered samples inconclusive when results differed in 2 different laboratories.

### Bioassay

Bank voles carrying isoleucine at the polymorphic PRNP codon 109 (Bv109I) were bred and inoculated at the Istituto Superiore di Sanità after approval of the experimental protocol from the Italian Ministry of Health (decree number 1122/2020-PR). We carried out all procedures in accordance with European Council directive 2010/63 and in compliance with the Italian Legislative Decree 26/2014. We inoculated 8-week-old Bv109I voles intracerebrally with 20 μL of 10% wt/vol tissue homogenates. We performed inoculations, clinical examinations, sampling, neuropathologic diagnosis, and WB analysis of PrP^Sc^ as previously described (*10*).

## Results

### Amplification of CWD Strains by PMCA Using Bank Vole Substrate

**Figure 1:**
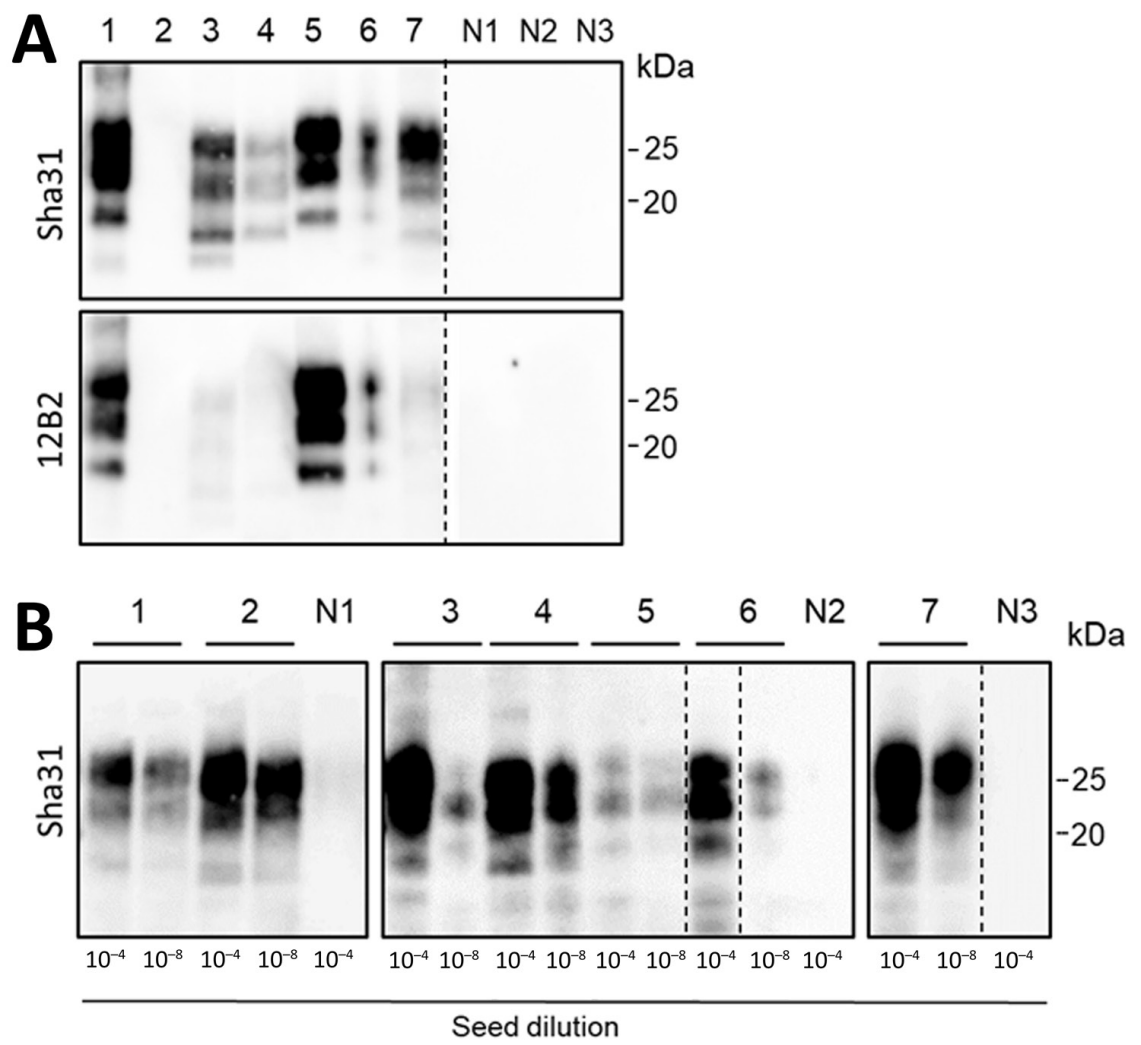
Western blots and protein misfolding cyclic amplification (PMCA; using bank vole substrate) of brain isolates of animals analyzed in a study of prions in muscles of cervids with chronic wasting disease, Norway. A) Western blot analyses of proteinase K–digested brain samples demonstrate the different PrP^Sc^ (misfolded forms of the prion protein) molecular profiles of the chronic wasting disease strains present in reindeer, moose (affected by different strains: N-terminally truncated PrP^Sc^ in moose A and B, and not N-terminally truncated PrP^Sc^ in moose C and D), and a red deer. The PrP^res^ (protease-resistant prion) signal visualized by Sha31 antibody demonstrates the typical electrophoretic migration of PrP^Sc^ protein bands, and the 12B2 antibody demonstrates the different cleavages at the N-terminal of the PrP (major prion) protein, which are characteristic for the different strains. B) PMCA amplification of the same brain samples (10^−4^ and 10^−8^ dilutions as indicated) using bank vole brain substrate. After 4 rounds of PMCA, amplicons were treated with proteinase K, and PrP^res^ was visualized by Western blot using Sha31 antibody. Our PMCA protocol efficiently amplified prions from all chronic wasting disease–affected samples, regardless of the different strains. No PrP^Sc^ amplification was observed in the brain of healthy negative control reindeer (N1), moose (N2), and red deer (N3). Lane designations: 1, reindeer A; 2, reindeer B; 3, moose A; 4, moose B; 5, moose C; 6, moose D; 7, red deer. In panel A, tissue equivalents of 10 mg were loaded for each lane, except lanes 3 (tissue equivalents: 2 mg) and 5 (tissue equivalents: 1 mg). The same quantities of PrP^res^ were loaded for both antibody visualizations. In panel B, equivalent sample volumes were loaded for all lanes. Numbers at the right indicate the molecular weight marker. Dashed lines between images depict membrane splicing.

WBs of brain homogenates of the animals used in this study confirmed the presence or absence of PrP^Sc^ in the materials. This analysis highlighted the presence of various types of PrP^res^ in relation to the known differences among species and within moose samples, using Sha31 antibodies, targeting the protein core, and 12B2 antibodies, targeting the N-terminal part of PrP protein (Figure 1, panel A). As expected, we detected PrP^res^ in the brain of all CWD-affected animals, with the exception of reindeer B, where we detected it only in the lymph node by ELISA. The WB analysis showed the typical molecular profile of PrP^res^ of the different strains, with banding patterns distinct across species, and among the moose with different genotypes. We observed a noticeable lower migration of the PrP^res^ bands and a substantial reduction of signal with the N-terminal antibody in moose A and B, as described in Pirisinu et al. (*13*), and in red deer, indicative of a truncation of the N terminus of PrP^res^ in these animals (Figure 1, panel A). In contrast to results for moose A and B, PrP^res^ in moose C and D were not N-terminal truncated (*17*). Those results confirmed the known PrP^Sc^ variability in CWD isolates from different cervid species in Norway and showed that the moose samples included in this study have 2 different PrP^Sc^ types.

We assessed the efficiency of the bank vole substrate in amplifying the Norway CWD prions by subjecting 2 dilutions (10^−4^ and 10^−8^, vol/vol) of infected brains to PMCA amplifications. We also included brain homogenates from healthy animals as negative controls. After 4 rounds of amplification, WB analysis of the products revealed PrP^res^ from all infected brain dilutions, indicating that the PMCA protocol used was successfully amplifying prions in reindeer, moose, and red deer, regardless of the CWD prion strains (Figure 1, panel B). We were unable to amplify PrP^Sc^ from the negative controls. Of interest, we also amplified PrP^Sc^ from the brain of reindeer B, an animal that had tested positive only in lymphoid tissue with traditional diagnostic tests.

### PrP^Sc^ Detected in Lymph Nodes and Peripheral Nerves of CWD-Affected Cervids

Our primary objective was to investigate the lymphatic dissemination of CWD prions in moose and red deer by PMCA. To assess the presence of potential tissue inhibitors in the amplification process, we spiked brain-derived CWD reindeer prions in tissue homogenates from healthy reindeer and then subjected them to PMCA. Results showed that spiked CWD prions in muscle and lymph node homogenates induced a similar amplification efficiency as those in brain homogenate (Appendix Figure 1), suggesting the lack of tissue-specific inhibitors in the samples.

**Figure 2:**
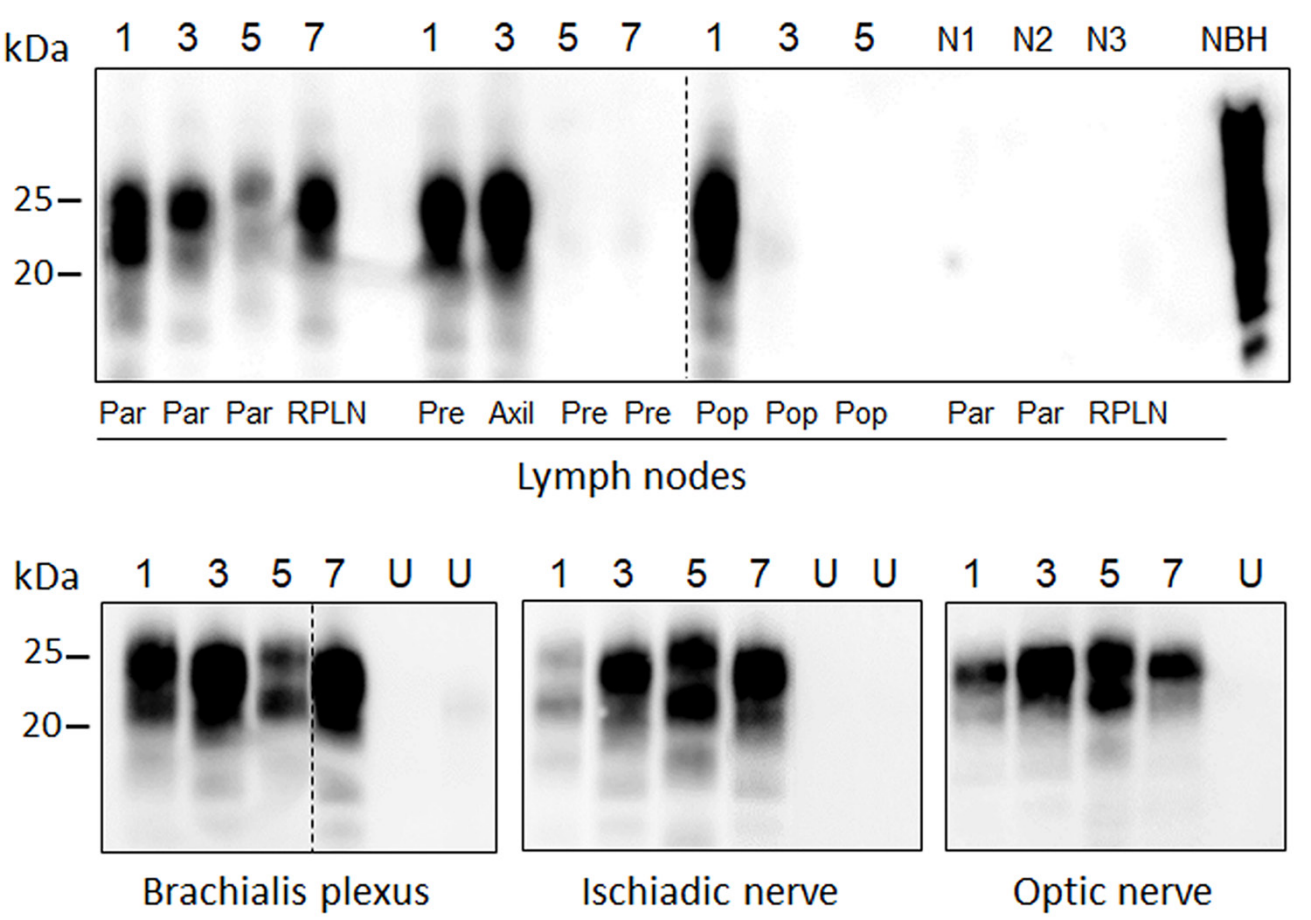
Protein misfolding cyclic amplification (PMCA) amplification of PrP^Sc^ (misfolded forms of the prion protein) in the lymphoreticular system and peripheral nerves of chronic wasting disease–affected animals from a study of prions in muscles of cervids with chronic wasting disease, Norway. We subjected lymph nodes and nerves collected from chronic wasting disease–affected cervids in Norway to serial PMCA before proteinase K digestion and Western blot analysis using Sha31 antibody. Example Western blots of samples from 1 representative animal of the different species and strains are shown; a more comprehensive result of all analyzed samples is summarized in Table 2. Lane designations: 1, reindeer A; 3, moose A; 5, moose C; 7, red deer. N lanes show lymph node from healthy, negative reindeer (N1), moose (N2), and red deer (N3). Dashed lines between images depict membrane splicing. Axil, axillary node; NBH, proteinase K–undigested bank vole brain homogenate used as electrophoretic migration marker of normal prion protein; par, parotid node; pre, prescapular node; pop, popliteal node; RPLN, retropharyngeal node; U, unseeded reaction included as a specificity control of PMCA reaction.

WB analysis of available lymph nodes collected from the head (parotid, retropharyngeal) or body (prescapular, axillary, and popliteal) before PMCA showed detection of PrP^res^ only in reindeer, and in none of the other samples (Appendix Figure 2, panel A). Strikingly, we also detected seeding activity in the red deer and moose after amplification. In those cases, we were able to detect PrP^res^ more readily in lymph nodes from the head than those located in the body (Table 2; Figure 2).

To examine the neural distribution of PrP^Sc^ in sites peripheral to the CNS, which was not detectable in moose and the red deer (Appendix Figure 2, panel B), we performed PMCA on the brachialis plexus, ischiadic nerve, and the optic nerve. The results showed prion amplification in all tested nerves (Figure 2).

### Detection of PrP^Sc^ Seeding Activity in Muscle Tissues of CWD-Affected Cervids

**Figure 3:**
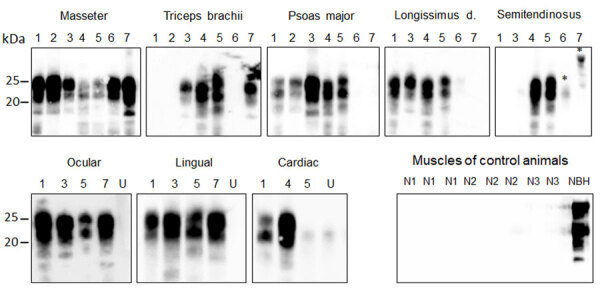
Detection of PrP^Sc^ (misfolded forms of the prion protein) amplification in muscle tissues of chronic wasting disease–affected cervids by protein misfolding cyclic amplification (PMCA) from a study of prions in muscles of cervids with chronic wasting disease, Norway. Muscle samples (10% homogenates) were subjected to 6 rounds of PMCA using bank vole brain homogenate substrate. PMCA products were treated with proteinase K before being analyzed by Western blot using Sha31 antibody to identify presence of PrP^Sc^. Results demonstrate efficient amplification of PrP^Sc^ in skeletal and cardiac muscles of chronic wasting disease–affected reindeer, moose, and a red deer. No PrP^Sc^ was amplified in negative control samples. Lane designations: 1, reindeer A; 2, reindeer B; 3, moose A; 4, moose B; 5, moose C; 6, moose D; 7, red deer. Numbers at left indicate the molecular weight marker. Asterisks indicate unspecific signals. NBH, proteinase K–undigested bank vole brain homogenate used as electrophoretic migration marker of normal prion protein (PrP^C^); U, unseeded reaction included as a specificity control of PMCA reaction.

Because of the popularity of venison in the human food chain, it is important to assess the presence of prions in the musculature of CWD-affected cervids. We examined 8 different muscles (Table 2), all of which were negative for PrP^res^ by direct WB (Appendix Figure 2, panel C). By PMCA, we amplified PrP^Sc^ from all muscle types tested, although with some individual variation (Table 2; Figure 3). Most muscles tested positive in reindeer and in 3 of 4 moose. We observed less positive PrP^Sc^ results in muscles from moose D and the red deer. Of the different muscles tested, masseter, ocular and lingual muscles from the head were always positive. In contrast, muscles located more peripherally from the head were negative in some animals. The semitendinosus muscle was the least frequently positive; only 2 of 6 samples tested positive. The efficiency of prion detection in skeletal muscles in moose, the red deer, and reindeer were similar in both laboratories, Ås and Milan, showing 92% agreement in the PMCA results (Appendix Table).

### Bioassay of Peripheral Tissues from Moose in Bank Voles

To determine whether prions amplified in peripheral tissues by PMCA are also infectious, we intracerebrally inoculated Bv109I animals with selected tissues (Table 3). We chose moose and red deer tissues because of the high susceptibility of Bv109I voles to CWD strains in those species (*10*,*18*), which we confirmed by the efficient transmission of the moose C brain homogenate (Table 3), compared with the very low susceptibility observed for reindeer CWD (*10*). Of the 7 peripheral tissues tested, the ocular muscle and parotideal lymph node from moose A transmitted the disease in Bv109I voles, but with a lower attack rate and longer survival time compared with the brain of the same moose (Table 3). We observed no evidence of infectivity in the popliteal lymph node and longissimus dorsi muscle of moose A, the prescapular lymph nodes of moose C and the red deer, and the longissimus dorsi muscle of moose C.

**Table 3:** Transmission in Bv109I voles of peripheral tissue from moose and red deer with CWD in a study of prions in muscles of cervids with CWD, Norway*

Animal	Inocula	Infectivity	
		Survival time†	Attack rate
Moose A	Brain‡	312 ± 46‡	13/13‡
	Parotid lymph node	561 ± 114	8/13
	Popliteal lymph node	>817	0/13
	Longissimus dorsi muscle	>944§	0/12
	Ocular muscle	494 ± 135¶	5/14
Moose C	Brain	150 ± 28	13/13
	Prescapular lymph node	>959	0/14
	Longissimus dorsi muscle	>936§	0/14
Red deer	Brain#	221 ± 36#	13/13#
	Prescapular lymph node	>1017	0/13

*Bv109I, bank voles carrying isoleucine at the polymorphic PRNP codon 109; CWD, chronic wasting disease. †Expressed as days postinoculation ± SD. ‡Transmission reported in Nono et al. (*10*) §Ongoing experiments; 3 inoculated Bv109I voles were healthy at the indicated days postinoculation. All other Bv109I voles were negative at postmortem. ¶Ongoing experiment; 4 inoculated Bv109I voles remained healthy at 932 days postinoculation. #Transmission reported in Marín-Moreno et al. (*18*).

## Discussion

Animals affected by CWD can remain asymptomatic for months, during which time prions can spread to various tissues and be shed into the environment, contributing to horizontal transmission (*1*). Depending on the prion strains, the PrP^Sc^ distribution pattern and tissue accumulation might differ in the host (*24*). Reports have identified 2 distinct patterns of prion spread in animal prion diseases (*6*,*27*–*30*). The first pattern involves the initial replication of prions in tissues from the lymphoreticular system, followed by spread to the peripheral nervous system before entering the brain. This pattern is observed in contagious strains, such as classical scrapie and North American CWD. The second pattern involves prion replication primarily in the CNS, as observed in less contagious or noncontagious prion diseases, such as BSE and Nor98/atypical scrapie.

There is limited research on the tissue distribution of the infectious agent in the newly emerging CWD strains in cervids in Norway. Through conventional diagnostic methods, investigators detected PrP^Sc^ in the lymph nodes of all reindeer with CWD but detected the prion in the CNS of only 50% of them. Conversely, researchers detected PrP^Sc^ in only the CNS in moose and the red deer (*13*,*15*). Using conventional WB in this study, we confirmed the presence of PrP^res^ in brain, lymph nodes, and nerves in reindeer, but only moose and the red deer demonstrated evidence of PrP^res^ in the brain (Figure 1, panel A; Appendix Figure 1). However, by using PMCA, we were able to demonstrate PrP^Sc^ seeding activity in all 3 species in various peripheral tissues, including the peripheral nerves, lymph nodes, and a variety of muscles. This finding illustrates the shortcomings of the conventional diagnostic methods in detecting minute quantities of prions, which impedes the accurate assessment of peripheral CWD distribution (*31*,*32*). Previous studies have demonstrated the presence of prions in multiple peripheral tissues and body fluids, including lymphoid tissues, peripheral nerves, muscles, blood, and excreta, in animals infected with North America CWD strains (*20*,*22*,*33*–*38*). The tissue distribution of PrP^Sc^ in reindeer, with a CWD strain similar to cases found in North America, was therefore not surprising. However, the findings of PrP^Sc^ in peripheral tissues in moose and red deer by PMCA were less expected, especially in muscles, given the sporadic occurrence and lack of evidence, to date, for contagiousness of these new CWD strains (*11*).

We confirmed the presence of prion infectivity in PMCA-positive lymphoid and muscle tissues by transmission in bank voles. Despite the limited number of samples examined, infectivity and seeding activity showed a good correlation: 2 of 4 positive transmissions from PMCA-positive samples and 0 of 3 from PMCA-negative samples. This correlation suggested PMCA to be a good proxy for CWD infectivity in bank voles, as observed in sheep scrapie (*39*). The negative transmission observed with 2 PMCA-positive tissues could be the result of the presence of very low levels of infectivity, below the detection limit of the vole bioassay. This finding is consistent with the partial transmission rate observed in the positive transmissions.

Identifying prions outside of CNS in cases of CWD in moose and the red deer mirrors the pattern observed in Nor98/atypical scrapie in sheep and atypical BSE forms in cattle, which represent additional sporadic animal prion diseases. Initially, researchers believed the accumulation of PrP^Sc^ to be confined to the CNS in these diseases (*27*). Nevertheless, subsequent studies using bioassay experiments demonstrated that infectivity could be detected in peripheral tissues that had been considered negative by traditional PrP^Sc^ detection techniques, including lymphoid tissues, nerves, and muscles (*40*,*41*).

Transmission studies have reported the presence of PrP^Sc^ in muscles in hamsters and sheep orally challenged with classical scrapie and, subsequently, in CWD-infected deer (*27*). Other studies reported detection of PrP^Sc^ in muscles of intracerebrally inoculated hamsters and mice, as well as in patients with Creutzfeldt-Jakob disease (*42*,*43*). In those instances, PrP^Sc^ may have reached the muscles via centrifugal spread, through peripheral nerves and the innervation of efferent and sensory nerve fibers to the tissues (*27*). Our investigation indicated that a similar phenomenon might occur in Norway strains, regardless of the cervid species involved. Of note, we observed a more efficient amplification of PrP^Sc^ in muscle and lymph node samples that were closer to the head and CNS compared with those further apart. Similarly, we detected infectivity in tissues from the head, the parotid lymph node, and the ocular muscle from moose A; however, the PMCA-positive popliteal lymph node and longissimus muscle from the same moose did not transmit in bank voles. Considering the incomplete transmission rate we observed in analyzing positive transmissions from peripheral tissues compared with the brain, we theorize that CWD prions accumulate at low titers in peripheral tissues and that prion titers might be higher in tissues closer to the CNS.

Unfortunately, in examining only field cases in our study, we concede that the lack of clinical status of the animals precludes establishing a correlation between the peripheral distribution of PrP^Sc^ and the disease stage. For instance, we were unable to determine whether the absence of seeding activity in some specific muscles and lymph nodes was a result of these animals being culled during early stages of the disease. We also could not rule out that the observed variation could be the result of very low prion titers in peripheral tissues or of individual strain characteristics.

In summary, the results of our study indicate that prions are widely distributed in peripheral and edible tissues of cervids in Norway, including muscles. This finding highlights the risk of human exposure to small amounts of prions through handling and consuming infected cervids. Nevertheless, we note that this study did not investigate the zoonotic potential of the Norway CWD prions. In North America, humans have historically consumed meat from CWD-infected animals, which has been documented to harbor prions (*35*,*44*–*47*). Despite the potential exposure to prions, no epidemiologic evidence indicates a correlation between the occurrence of CWD cases in animals and the prevalence of human prion diseases (*48*). A recent bioassay study reported no transmissions from 3 Nordic isolates into transgenic mice expressing human PrP (*49*). Therefore, our findings should be interpreted with caution in terms of human health implications, and further research is required to determine the zoonotic potential of these CWD strains.

The presence of prions in peripheral tissues indicates that CWD may have a systemic nature in all Norwegian cervid species, challenging the view that prions are exclusively localized in the CNS in sporadic CWD of moose and red deer. Our findings expand the notion of just how widely distributed prions can be in cervids affected with CWD and call into question the capability of emerging CWD strains in terms of infectivity to other species, including humans.

Appendix Additional information for prions in muscles of cervids with chronic wasting disease, Norway
