# Supplementary material for: Prions in Muscles of Cervids with Chronic Wasting Disease, Norway
Source: Emerg Infect Dis. 2025 Feb;31(2):246–55. doi: 10.3201/eid3102.240903 (PMC11845164; doi:10.3201/eid3102.240903)
Supplement: Appendix — Additional information for prions in muscles of cervids with chronic wasting disease, Norway [file 24-0903-Techapp-s1.pdf]

Article DOI: http://doi.org/10.3201/eid3102.240903

EID cannot ensure accessibility for supplementary materials supplied by authors. Readers who have difficulty accessing supplementary content should contact the authors for assistance.

Prions in Muscles of Cervids with Chronic Wasting Disease, Norway

Appendix

Appendix Table. Summary of PMCA amplification results obtained from samples that were independently analyzed in the laboratory in Ås (Lab-Å) and in Milan (Lab-M).

		Lab-Å/Lab-M						
Tissues		**Reindeer A**	**Reindeer B**	**Moose A**	**Moose B**	**Moose C**	**Moose D**	**Red deer**
Lymph nodes	Parotid			+/+				
	RpLN					+/+		
	Mandibular						−/−	
	Prescapular						**−/+**	−/−
	Axillary			+/+				
	Popliteal						−/−	
Nerves	Ischiadic			+/+			+/+	
	Brachial plexus			+/+				
Muscle	Masseter	+/+	+/+	+/+	+/+	+/+	+/+	+/+
	Triceps brachii	**−/+**	−/−	+/+	+/+	+/+	**−/+**	+/+
	Psoas major	+/+	+/+	+/+	+/+	+/+	−/−	−/−
	Longissimus dorsi	−/−		+/+	+/+	+/+	−/−	**−/+**
	Semitendinosus	−/−		−/−	+/+	+/+	−/−	−/−

Boldface indicates discrepant results between the 2 laboratories.

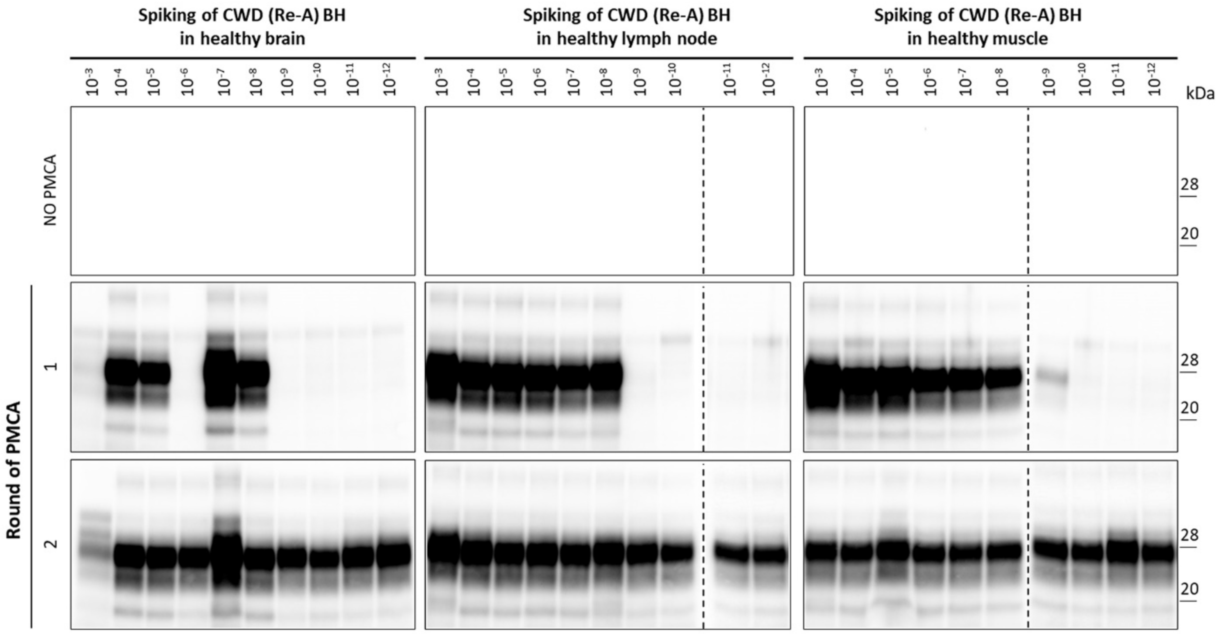

Appendix Figure 1. Assessment of potential tissue inhibitors in PMCA analysis. CWD-affected reindeer brain homogenate (BH) was spiked (from 10^{-3} to 10^{-12}) in healthy brain, muscle, and lymph node.

Samples were subjected to PMCA with bank vole substrate and PrP^{res} signal was detected by Western blot after proteinase K digestion using 6D11 antibody. Two rounds of amplification allowed detection of PrP^{res} in all dilutions of all tissues, indicating similar amplification efficiency among tissues. Numbers at right indicate the molecular weight marker. Dashed lines between images depict membrane splicing.

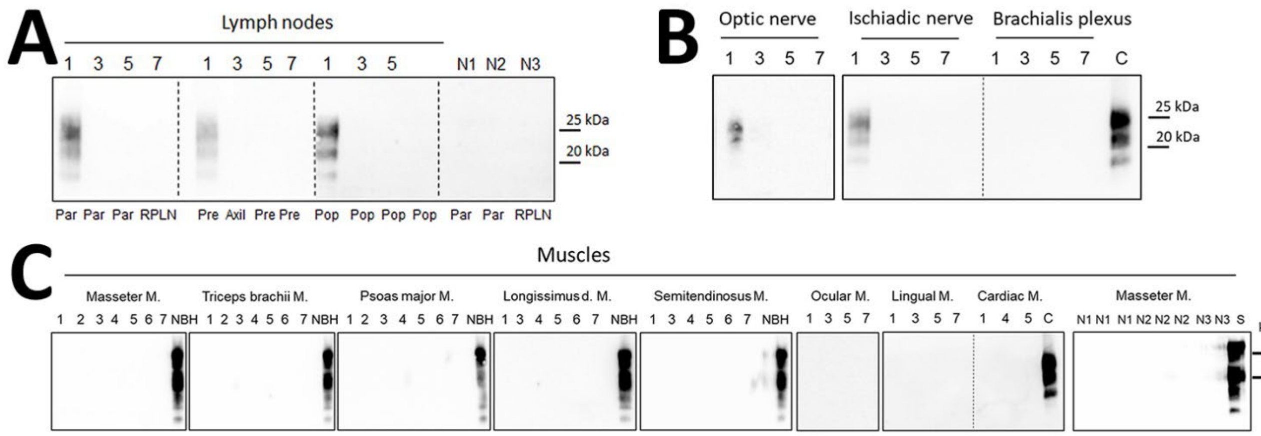

Appendix Figure 2. Western blot (WB) analysis of tissue samples used in this study to show the presence/absence of PrP^{Sc} before amplifying by PMCA. Tissue homogenates (10% w/v) of A) lymph nodes (Par, parotid; RPLN, retropharyngeal; Axil, axillary; Pre, prescapular and Pop, popliteal), B) nerves, and C) muscles were treated with 100 µg/mL proteinase K at 37°C for 1 hour. Digestion was stopped by adding Laemmli sample buffer and boiled at 100°C for 5 minutes before SDS-PAGE analysis using NuPAGE 12% Bis-Tris protein gels (Thermo Scientific, https://www.thermofisher.com). Proteins were transferred onto a PVDF membrane and PrP^{res} signals were detected using sha31 antibody. Results showed PrP^{res} in the lymph nodes and the peripheral nerves (but not in muscle) of reindeer, and no detectable PrP^{res} in any tissue samples of moose and red deer that previously tested negative by diagnostic methods. Lanes 1, reindeer A; 2, reindeer B; 3, moose A; 4, moose B; 5, moose C; 6, moose D; 7, red deer. N1–3, lymph node from healthy negative reindeer, moose, and red deer, respectively. NBH, PK-undigested bank vole brain homogenate used as electrophoretic migration marker of normal prion protein (PrP^{C}). C, proteinase K–digested brain material from scrapie-infected sheep included as WB control.
